# Host Mesothelin Expression Increases Ovarian Cancer Metastasis in the Peritoneal Microenvironment

**DOI:** 10.3390/ijms222212443

**Published:** 2021-11-18

**Authors:** Tyvette S. Hilliard, Brooke Kowalski, Kyle Iwamoto, Elizabeth A. Agadi, Yueying Liu, Jing Yang, Marwa Asem, Yuliya Klymenko, Jeff Johnson, Zonggao Shi, Gifty Marfowaa, Madeleine G. Yemc, Phillip Petrasko, M. Sharon Stack

**Affiliations:** 1 Department of Chemistry and Biochemistry, University of Notre Dame, Notre Dame, IN 46556, USA; brooke.l.kowalski.51@alumni.nd.edu (B.K.); loughran.11@nd.edu (E.A.A.); masem.88.ma@gmail.com (M.A.); sstack@nd.edu (M.S.S.); 2 Harper Cancer Research Institute, University of Notre Dame, Notre Dame, IN 46556, USA; yliu12@nd.edu (Y.L.); jyang@nd.edu (J.Y.); yuliya.klymenko.1@nd.edu (Y.K.); jjohns39@nd.edu (J.J.); 3 Department of Chemical and Biomolecular Engineering, University of Notre Dame, Notre Dame, IN 46556, USA; kyle.m.iwamoto.1@alumni.nd.edu; 4 Integrated Biomedical Sciences Graduate Program, University of Notre Dame, Notre Dame, IN 46556, USA; 5 Department of Biological Sciences, University of Notre Dame, Notre Dame, IN 46556, USA; 6 St. Jude Children’s Research Hospital, Memphis, TN 38105, USA; Shizonggao@gmail.com; 7 Department of Pre-Professional Studies, University of Notre Dame, Notre Dame, IN 46556, USA; gmarfowa@alumni.nd.edu; 8 Department of Science Business, University of Notre Dame, Notre Dame, IN 46556, USA; Myemc@alumni.nd.edu (M.G.Y.); ppetrask@alumni.nd.edu (P.P.)

**Keywords:** ovarian cancer, metastasis, mesothelium, mesothelin, cell adhesion, collagen, multicellular aggregates

## Abstract

Mesothelin (MSLN), a glycoprotein normally expressed by mesothelial cells, is overexpressed in ovarian cancer (OvCa) suggesting a role in tumor progression, although the biological function is not fully understood. OvCa has a high mortality rate due to diagnosis at advanced stage disease with intraperitoneal metastasis. Tumor cells detach from the primary tumor as single cells or multicellular aggregates (MCAs) and attach to the mesothelium of organs within the peritoneal cavity producing widely disseminated secondary lesions. To investigate the role of host MSLN in the peritoneal cavity we used a mouse model with a null mutation in the MSLN gene (MSLN^KO^). The deletion of host MSLN expression modified the peritoneal ultrastructure resulting in abnormal mesothelial cell surface architecture and altered omental collagen fibril organization. Co-culture of murine OvCa cells with primary mesothelial cells regardless of MSLN expression formed compact MCAs. However, co-culture with MSLN^KO^ mesothelial cells resulted in smaller MCAs. An allograft tumor study, using wild-type mice (MSLN^WT^) or MSLN^KO^ mice injected intraperitoneally with murine OvCa cells demonstrated a significant decrease in peritoneal metastatic tumor burden in MSLN^KO^ mice compared to MSLN^WT^ mice. Together, these data support a role for host MSLN in the progression of OvCa metastasis.

## 1. Introduction

Mesothelin (MSLN) is a glycosyl phosphatidylinositol (GPI)-anchored glycoprotein synthesized as a 70 kDa precursor that is proteolytically cleaved by furin, resulting in the 40 kDa MSLN fragment and a 30 kDa megakaryocyte potentiating factor fragment. Normally, MSLN is expressed at low levels in mesothelial cells that line the peritoneum, pleura and pericardium. Conversely, MSLN is known to be overexpressed in several human cancers including mesotheliomas, ovarian, pancreatic and gastric. Interestingly, these cancers have a strong preference to metastasize to the peritoneal cavity [1,2,3,4,5,6]. Furthermore, MSLN has been identified as a potential tumor-associated marker in approximately 70% of ovarian cancers [1,6,7,8]. MSLN expression has been correlated with alterations in cell survival, proliferation, adhesion, and overall tumor progression [2,9]. High MSLN expression in human cancer cell lines, including ovarian cancer, has been associated with tumor cell adhesion, increased tumor burden, invasion through mesothelial cells, dissemination within the peritoneal cavity and chemoresistance [1,9,10,11,12]. While tumor-naïve MSLN knockout mice do not present with an abnormal phenotype [13], the contribution of host MSLN expression to peritoneal tumor dissemination has not been explored.

Ovarian cancer (OvCa) is the most lethal gynecological malignancy among US women and the second most common cause of gynecologic cancer death worldwide. The major cause of death is due to metastasis from the primary tumor to organs within the peritoneal cavity. Women diagnosed with ovarian cancer have an overall 5-year survival rate of 47%, however, women diagnosed with advanced-stage disease, with substantial intraperitoneal metastasis, have a 5-year survival rate of only 29% [14,15]. OvCa primarily metastasizes by the shedding of single tumor cells or multicellular aggregates (MCAs), from the primary tumor. With the assistance of natural fluid flow, the metastatic cells and MCAs circulate throughout the peritoneal cavity and attach to the mesothelial lining of peritoneal organs, including but not limited to the abdominal peritoneum, and omentum [16,17,18]. As OvCa progresses to advanced-stage disease, malignant ascites fluid accumulates in the peritoneal cavity due to impaired lymphatic fluid drainage, vascular permeability, and increased net infiltration, significantly contributing to poor quality of life and eventually mortality [19,20]. Additionally, it was reported that OvCa patients have a higher concentration of MSLN in peritoneal ascites fluid when compared to patients with benign tumors [21].

Organs in the abdominal cavity, including but not limited to the peritoneum and omentum are covered by the mesothelium, a single layer of mesothelial cells covering a basement membrane composed of collagen I and IV, fibronectin and laminin. The mesothelium forms a protective barrier to the abdominal organs and supports the homeostasis of the abdominal cavity [22,23]. Adhesion of ovarian cancer cells to mesothelial cells in the peritoneal cavity is a key early event in ovarian cancer metastasis. Additionally, mesothelial cells have been associated with the promotion of ovarian tumor cell adhesion, proliferation and invasion [23]. Although MSLN and mesothelial cells have been implicated in ovarian cancer progression in several studies, the potential contribution of host MSLN expression to the metastatic success of ovarian cancer has not yet been addressed [1,9,10,12].

In the current study, we investigate the role of host MSLN expression in normal peritoneal tissues, MCA formation and ovarian cancer metastasis using an MSLN wild-type (MSLN^WT^) and knockout (MSLN^KO^) mouse model. Our results show that deletion of host MSLN expression alters the ultrastructure of tumor susceptible host tissues, decreases MCA size and significantly decreases intraperitoneal ovarian tumor burden. Overall, deletion of host MSLN expression resulted in a less favorable microenvironment for ovarian tumor cell adhesion and metastatic anchoring.

## 2. Results

### 2.1. Host MSLN Expression in Wild Type and Knockout Mesothelial Cells

**Figure 1:**
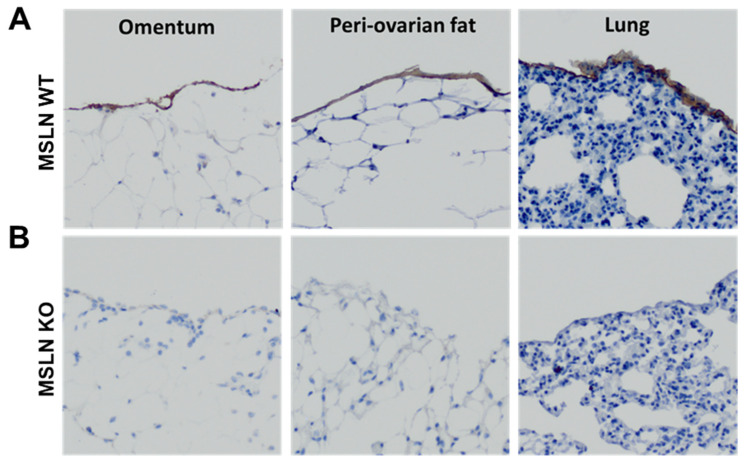
Immunohistochemical detection of MSLN. Normal omentum, peri-ovarian fat, and lung tissues were dissected from (**A**) MSLN^WT^ and (**B**) MSLN^KO^ and were immunohistochemically stained for mesothelin. Expression was confirmed in MSLN^WT^ mice and absent in MSLN^KO^ mice. Images taken at 20×.

Under normal conditions, MSLN is expressed in the mesothelium-lined tissues of the pericardial, peritoneal and pleural cavities. To verify the deletion of host MSLN, mice were genotyped using DNA collected from ear punches. PCR was performed and the product was electrophoresed on a 1% agarose gel. Mice genotyped as wild type and homozygous knockout were used (data not shown). Immunohistochemical analysis, using an anti-mesothelin antibody, was performed to visualize the expression of MSLN in the monolayer of mesothelial cells of tissues in the peritoneal (omentum and peri-ovarian fat) and pleural (lung) cavities of MSLN^WT^ and MSLN^KO^ mice. Whereas MSLN expression was observed in the mesothelial monolayer of omentum, peri-ovarian fat and lung tissues of MSLN^WT^ mice (Figure 1A), no expression was observed in MSLN^KO^ mice (Figure 1B), verifying knockout of MSLN.

### 2.2. Alterations in the Peritoneal Ultrastructure of MSLN^KO^ Mice

**Figure 2:**
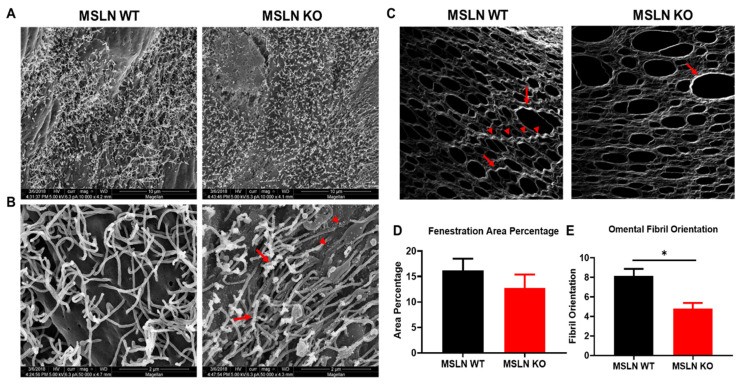
Ultrastructural analysis of MSLN^WT^ and MSLN^KO^ peritoneal tissues. Peritoneum of MSLN^WT^ and MSLN^KO^ mice were imaged using a scanning electron microscope. Images of mesothelial cells were taken at (**A**) 10,000× and (**B**) 50,000×. MSLN^KO^ peritoneum displayed structural differences in microvilli including rough cell surface, underdeveloped microvilli nanoscale nodular structure (arrow heads), and excessive budding nodules (arrows) from microvilli compared to MSLN^WT^ peritoneum. (**C**) Evaluation of omental collagen by second harmonic generation (SHG) microscopy. Representative images of omental collagen from MSLN^WT^ and MSLN^KO^ mice. Long (arrow heads) and thick (arrows) banding were more prominent in MSLN^WT^ tissues. MSLN^KO^ tissues have less thick banding (arrow). (**D**) Using ImageJ, fenestration area percentage and (**E**) fibril orientation was measured using SHG images. * *p* < 0.05; error bars represent standard error of mean.

Ovarian cancer cell attachment to mesothelial cells is an essential early event in metastatic dissemination and surface microvilli have been implicated in this process [24]. Scanning electron microscopy (SEM) was used to visualize the peritoneal mesothelial cell surface in MSLN^WT^ and MSLN^KO^ mice. At low resolution (10,000× magnification), the surface distribution of microvilli appeared unaltered by MSLN deletion (Figure 2A). In contrast, high-resolution SEM imaging (50,000× magnification) showed abnormal microvilli on MSLN^KO^ mesothelial cells, with truncated length and an increase in nanoscale nodular structures on both the mesothelial cell surface (Figure 2B; red arrowheads) and microvilli (Figure 2B; red arrows) relative to microvilli on MSLN^WT^ mesothelial cells.

After adhesion to mesothelial cells, ovarian tumor cells penetrate the mesothelial monolayer and cause mesothelial cell retraction, exposing the collagen-rich sub-mesothelial matrix into which cells migrate, anchor and proliferate to generate secondary lesions. Second-harmonic generation microscopy was used to visualize the collagen quaternary structure of tumor naïve omental tissues (Figure 2C). MSLN^WT^ omental tissues displayed collagen that had more prominent long (Figure 2C; arrow heads) and thick banding (Figure 2C; arrow) compared to MSLN^KO^ tissues. MSLN^KO^ tissues exhibited a more robust meshwork between fenestrations (openings) when compared to MSLN^WT^ omental tissues. Similarly, when quantified, fenestrations covered less area in MSLN^KO^ omental tissues than MSLN^WT^ omental tissues however this difference was not significant (Figure 2D). To quantify the collagen fibrillar structures in omental tissues, the ImageJ-based FibrilTool was used to quantify the average fibril orientation of the collagen fibers. The fibril orientation corresponds to the average angle of collagen fibrils in tissues. Omental tissues from MSLN^KO^ mice displayed less oriented collagen fibrils compared to MSLN^WT^ mice (Figure 2E).

### 2.3. Host MSLN Expression Regulates In Vitro Multicellular Aggregate Size

**Figure 3:**
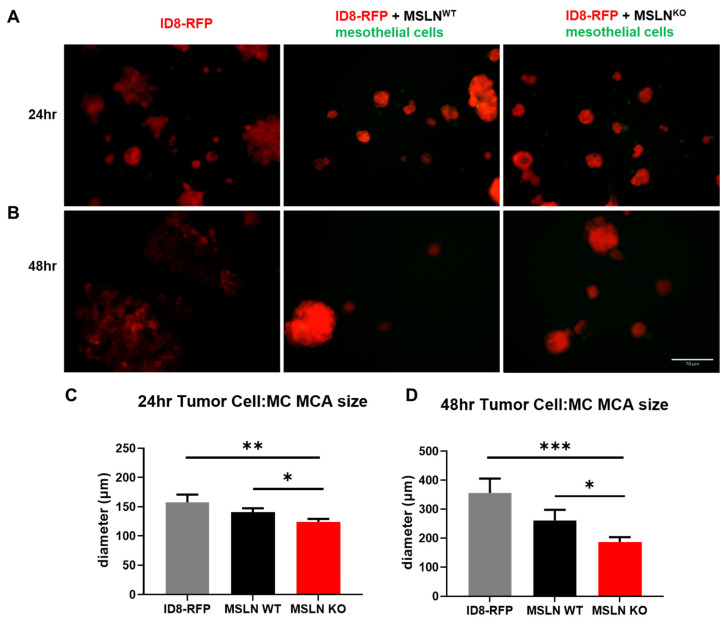
Multicellular aggregate co-culture with ovarian cancer cell line and MSLN^WT^ or MSLN^KO^ mesothelial cells. (**A**,**B**) ID8 ovarian cancer cells tagged with RFP were cultured with or without green CMFDA labeled primary mesothelial cells isolated from 3–6 month aged MSLN^WT^ and MSLN^KO^ mice for (**A**) 24 h and (**B**) 48 h. Compact multicellular aggregates were formed in the presence of mesothelial cells regardless of MSLN expression. Multicellular aggregates formed with MSLN^WT^ mesothelial cells were significantly larger after (**C**) 24 h and (**D**) 48 h. Scale bar = 70 µm. * *p* < 0.05, ** *p* < 0.005, *** *p* <0.001.

Recent reports indicate MCAs found in ascites fluid are heterotypic, harboring both tumor cells and mesothelial cells [25,26]. To recapitulate the cellular diversity of MCAs found in human ascites fluid, the hanging drop method was used to create MCAs with RFP labeled ID8 murine ovarian cancer cells grown in the presence or absence of green CMFDA dyed MSLN^WT^ or MSLN^KO^ mesothelial cells. ID8-RFP cells alone grew large MCAs after 24 h (Figure 3A) and by 48 h the cells started to attach to the tissue culture lid and grow as a monolayer (Figure 3B). Tumor cells in the presence of mesothelial cells, regardless of MSLN expression, facilitated MCA formation demonstrated by tightly packed spheroids (Figure 3A,B). However, the MCAs with MSLN^WT^ mesothelial cells grew significantly larger than MCAs with MSLN^KO^ mesothelial cells after 24 h (Figure 3A,C) and 48 h (Figure 3B,D). Additionally, after 24 h, there appeared to be more MSLN^KO^ mesothelial cells not associated with the cancer cells than MSLN^WT^ mesothelial cells (Figure 3A; green cells).

### 2.4. Lack of Host MSLN Expression Alters In Vivo Adhesion to Abdominal Adipose

**Figure 4:**
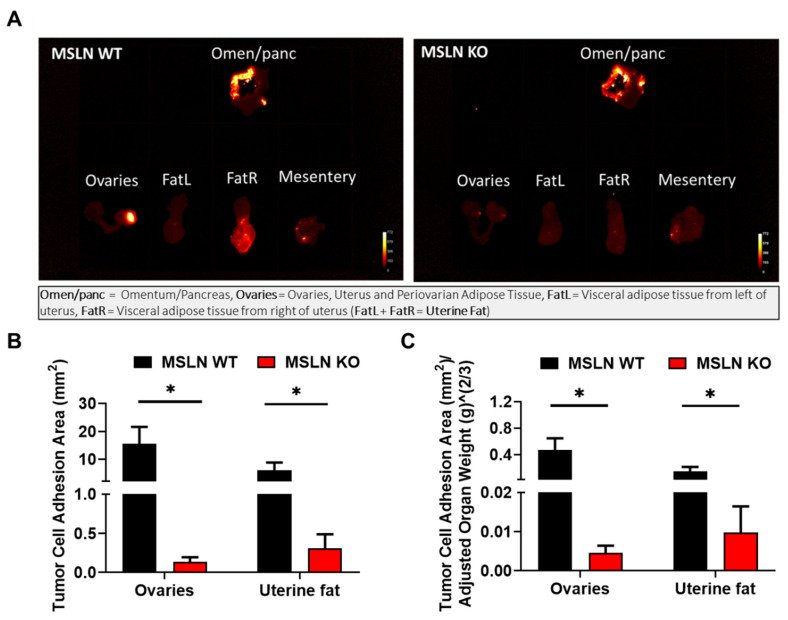
In vivo adhesion assay. (**A**) 3–6 month aged MSLN^WT^ and MSLN^KO^ mice were injected i.p. with 10 × 10^6^ ID8 ovarian cancer cells tagged with RFP. Mice were sacrificed the next day and imaged to determine short-term cellular adhesion in adipocyte-rich tissues. (**B**) Cellular adhesion was quantified with ImageJ by the Tumor Cell Adhesion Area (area of the organ occupied by cell adhesion) and (**C**) Weight Adjusted Tumor Cell Adhesion Area (area of the organ occupied by cell adhesion divided by scale-adjusted weight of the organ). MSLN^KO^ mice had significantly less cellular adhesion in the ovaries and the uterine fat (FatL + FatR). *n* = 5 for both MSLN^WT^ and MSLN^KO^; * *p* < 0.05; error bars represent standard error of mean.

To investigate whether host MSLN expression impacts early events in metastatic dissemination, an in vivo adhesion assay was performed. MSLN^WT^ and MSLN^KO^ mice were injected intraperitoneally (i.p.) with ID8-RFP cells and sacrificed after ~18 h. Peritoneal adipose depots, known to be the initial sites of metastatic dissemination, were excised and imaged (Figure 4A). Results show a significant reduction in tumor cell adhesion to peri-ovarian and uterine adipose in MSLN^KO^ mice relative to MSLN^WT^ mice (Figure 4B,C). Interestingly, there was no difference in ovarian tumor cell adhesion to omental adipose of MSLN^WT^ and MSLN^KO^ mice (quantification not shown), suggesting that factors other than host MSLN expression influence early omental seeding.

### 2.5. Deletion of Host MSLN Impacts Tumor Metastasis and Peritoneal Dissemination

**Figure 5:**
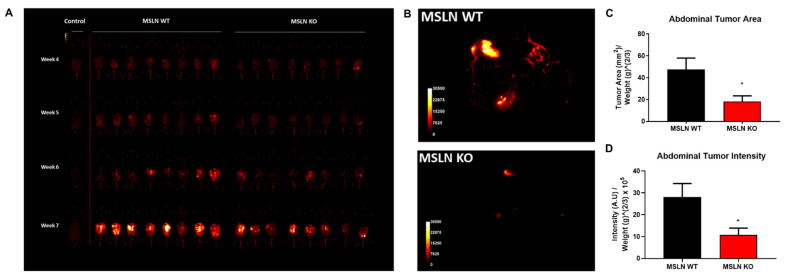
In vivo and in situ tumor burden of MSLN^WT^ and MSLN^KO^ mice. (**A**) 3–6 month aged cohorts were injected i.p. with 7 × 10^6^ ID8 ovarian cancer cells tagged with RFP. Mice were imaged under anesthesia at 4-, 5-, 6- and 7-weeks post-injection to monitor tumor progression using a Bruker Xtreme In Vivo imaging system. The live imaging data suggested MSLN^KO^ mice had less tumor burden compared with MSLN^WT^ mice. (**B**) At 8 weeks post-injection, end point dissection was performed. The abdominal cavity was exposed and the entire body was imaged using the Bruker Xtreme In vivo imaging system. (**C**) Abdominal tumor burden was quantified with ImageJ using two parallel methods of analysis. Abdominal Tumor Area (the tumor area divided by a scale-adjusted weight of the animal) and (**D**) Abdominal Tumor Intensity (the fluorescence intensity of the tumors divided by a scale-adjusted weight of the animal) both demonstrated that MSLN^KO^ mice develop significantly less tumor burden than MSLN^WT^ mice. *n* = 8 for both MSLN^WT^ and MSLN^KO^; * *p* < 0.05; error bars represent standard error of mean.

**Figure 6:**
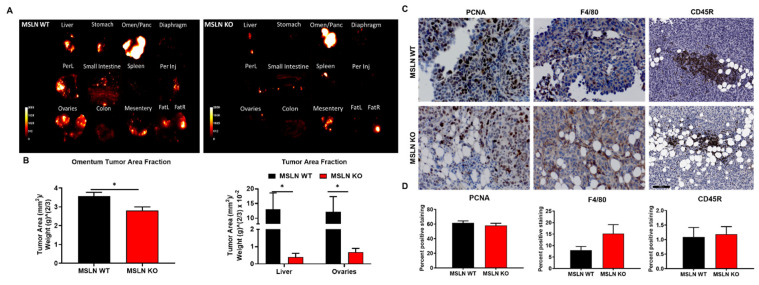
Organ-specific tumor burden. (**A**) Individual organs were dissected and imaged. (**B**) Tumor burden was quantified with ImageJ. Among all abdominal organs, the omentum, liver and ovaries from MSLN^KO^ mice had significantly less tumor burden than MSLN^WT^ mice. * *p* < 0.05; error bars represent standard error of mean. (**C**) Immunohistochemical analysis of omental tumor tissues. Omental tumor tissue IHC analysis of proliferation (PCNA), macrophages (F4/80), and tumor infiltrating lymphocytes (CD45R). Representative images of MSLN^WT^ and MSLN^KO^ omental metastases are shown. Scale bar = 100 µm. (**D**) Quantitation of positive staining. Analysis was carried out with the Aperio Image Analysis Tools package. Error bars represent standard error of mean. Omen/panc = Omentum/Pancreas, PerL = Parietal Peritoneum Left, Per Inj = Parietal Peritoneum Injection Side, Ovaries = Ovaries/Uterus and Periovarian Adipose Tissue, FatL = Visceral adipose tissue from left of uterus, FatR = Visceral adipose tissue from right of uterus.

To explore whether host MSLN expression affects overall metastatic tumor burden, MSLN^WT^ and MSLN^KO^ mice were injected i.p. with ID8-RFP murine ovarian cancer cells and tumor progression was monitored via longitudinal in vivo imaging beginning 4 weeks post-injection (Figure 5A). At 8 weeks post-injection, mice were euthanized, imaged, and abdominal tumor area and intensity were quantified. MSLN^KO^ mice displayed significantly less overall metastatic tumor burden relative to MSLN^WT^ mice (2.6-fold reduction, Figure 5B–D). To quantify organ-specific tumor burden, individual organs were dissected, imaged and analyzed. Notably, the majority of metastatic tumor burden was in the omentum (Figure 6A,B). Furthermore, the omentum, ovaries and liver from MSLN^KO^ mice demonstrated a significant decrease in tumor burden when compared to MSLN^WT^ mice (Figure 6A,B).

Immunohistochemical staining of formalin-fixed metastatic omental tumor tissues was used to examine the proliferation and the infiltration of lymphocytes and macrophages all of which may influence the decrease in tumor burden observed in MSLN^KO^ mice. To determine if differences in tumor burden were a result of proliferating tumor cells, a proliferating cell nuclear antigen (PCNA) antibody was used on omental metastases, however, no significant difference in proliferating tumor cells colonizing MSLN^WT^ and MSLN^KO^ omental metastases was observed (Figure 6C,D). To further explore how host MSLN expression impacts tumor burden, we analyzed omental metastases for tumor-infiltrating lymphocytes (TILs) and macrophages using a pan-b cell marker (CD45R) and pan macrophage (F4/80) marker. There were no significant differences in TIL staining between MSLN^WT^ and MSLN^KO^ omental metastases observed. Although there was an increase in macrophage infiltration in MSLN^KO^ tissues, the difference was not statistically significant (Figure 6C,D). 

## 3. Discussion

Mice with a null mutation in the mesothelin gene do not exhibit any phenotypic abnormalities compared to their wild-type littermates [13]. However, ultrastructural analysis of the mesothelial cell surface identified abnormal, nodular microvilli on the surface of MSLN^KO^ mesothelial cells. Mesothelial cells are the first line of defense to metastasizing cancer cells, providing a frictionless protective barrier between the abdominal organs [27]. Mesothelial cells are in constant contact with the normal peritoneal fluid that is low in protein concentration [28]. It is thought that the deletion of MSLN could possibly cause an influx of proteins that are functionally similar to MSLN, however, there are no reports in the literature that identify such proteins [13]. Albeit, an influx of proteins in the peritoneal cavity could explain the abnormal microvilli and small nanoscale nodular structures seen in the MSLN^KO^ mice. Madison et al. demonstrated peritoneal mesothelial cells have a smooth cell surface with varying microvilli density under normal conditions, however, an increase in protein concentration in the peritoneal cavity resulted in a rippled cell surface, underdeveloped microvilli nodules, and microvilli with abnormal nodular outgrowths, nevertheless the function of these microvilli nodular structures is still unknown [28]. Additionally, prominin-1 (prom-1), a cell surface biomarker known as an organizer of cellular membrane protrusions and a cell surface marker of cancer stem cells has been associated with ovarian cancer and other solid tumors [25,29]. Normal expression of prom-1, results in an increase in the number of microvilli, whereas mutated prom-1 expression results in a knob-like or branched morphology [30]. We similarly demonstrate ultrastructural differences in peritoneum microvilli including a rippled cell surface, abnormal microvilli with numerous microvilli outgrowths and underdeveloped microvilli nanoscale nodular structures in MSLN^KO^ mice. Together these data suggest that deletion of host MSLN may alter protein homeostasis in the peritoneal cavity. Although interesting, the identification of these potentially altered protein(s) and elucidation of their function(s) is outside the scope of the current study.

After initial adhesion, tumor cells invade the sub-mesothelial matrix and proliferate to create secondary metastatic sites. In the current study, ultrastructural analysis of the collagen-rich sub-mesothelial matrix indicated a higher degree of fibril orientation in MSLN^WT^ omental tissues relative to MSLN^KO^ tissues, contributing to the observation of long, thick collagen bands in omental tissue from MSLN^WT^ mice. The thick banding of collagen fibers is a result of collagen crosslinks that contribute to collagen orientation and subsequent mechanical properties of the omental tissues [31,32]. Studies have shown that an increase in the alignment of collagen fibers promotes cell migration and subsequently an increase in metastasis [33,34]. Tumor cells have been shown to realign less organized collagen fibers to facilitate invasion [35]. Furthermore, collagen fibril organization pathways have been shown to be enriched in metastatic high-grade serous ovarian cancer [36]. Additionally, MSLN^WT^ omental tissues display more fenestrations posing a lower physical barrier and allowing for more tumor cell invasion.

Ascites fluid accumulation containing multicellular aggregates within the peritoneal cavity is a hallmark of advanced-stage ovarian cancer. MCA formation is a hallmark of cancer stem cells [37]. As key mediators of metastasis, MCAs found in ascites fluid can range in sizes between 50 and 750 µm [38]. MCAs found in ascites can be both loose and tightly packed aggregates [39]. Compact MCAs are associated with a greater capacity for migration, invasion and survival [40,41,42]. Furthermore, MCA formation protects tumor cells against chemotherapeutics allowing for chemoresistance or recurrence [42]. We show that MCA compactness is associated with the co-culture of mesothelial cells with ovarian tumor cells suggesting the importance of mesothelial cells in compact MCA formation regardless of MSLN expression. However, MSLN expression does regulate MCA size, as mesothelial cells that express MSLN produce larger MCAs when co-cultured with ovarian cancer cells. As shown by others, MCA compactness is evident of a more aggressive and invasive subpopulation of ovarian tumor cells [41,42,43]. Differences in MCA size were observed in human ascitic fluid and may be the result of detachment or the preservation of proliferation from the primary tumor [41]. A larger spheroid size was observed in a study of the spheroid formation using a co-culture of several human ovarian tumor cell lines with rat mesothelial cells. These large spheroids demonstrated proliferating tumor cells in the peripheral zone but low proliferation in the inner zone of the spheroid where the mesothelial cells were located [25]. Moreover, larger MCAs were also shown to have higher drug resistance compared to smaller MCAs [44].

Peritoneal metastasis is commonly observed in ovarian cancer and is the key cause of a poor prognosis and an unfavorable outcome in patients. Normally, MSLN is expressed by mesothelial cells in the pleura, pericardium, and peritoneum and in the epithelial surface of the ovary and fallopian tubes [45]. However, MSLN is overexpressed in human ovarian tumors and correlates to poor survival [10]. When human OvCa cell lines were genetically engineered to have a gain of function to MSLN, there was an increase in tumor cell survival and invasion both in vitro and in vivo and the opposite was observed in loss of function cells suggesting the importance of MSLN expression in tumor cells [12]. Conversely, for the first time, we investigate the impact of host MSLN expression in ovarian cancer metastasis. When ovarian tumor cells shed from the primary tumor and disseminate throughout the peritoneal cavity, they adhere to the mesothelial cell layer of peritoneal organs. To assess early-stage adhesion events, we injected RFP-tagged ovarian tumor cells i.p. in our MSLN mouse model. Allowing time for cells to adhere, but not long enough to generate tumors, demonstrated initial homing of ovarian tumor cells to the omentum in both MSLN^WT^ and MSLN^KO^ mice. Deletion of host MSLN expression decreased ovarian tumor cell adhesion to peri-ovarian and uterine adipose suggesting a role of host MSLN expression in initial tumor cell adhesion. The reduction of early-stage adhesion could be due to the loss of CA125:MSLN binding. The ovarian cancer antigen CA125 was identified as a MSLN ligand that mediates heterotypic cellular adhesion, therefore allowing CA125 expressing tumor cells to bind to MSLN expressing mesothelial cells that line organs in the peritoneal cavity such as the peritoneum or omentum [3,8,9,46].

To recapitulate advanced stage OvCa with peritoneal metastasis and ascites accumulation, we utilized a syngeneic immunocompetent mouse model of advanced-stage ovarian cancer metastasis, to investigate the role of host MSLN expression on ovarian cancer metastasis [47,48,49,50]. Overall abdominal tumor burden was significantly decreased in MSLN^KO^ mice, most notably impacting metastatic colonization of omental, ovary and liver tissues, suggesting host MSLN expression is important in the metastatic success of ovarian tumor cells and in the progression of the disease. Interestingly, however, host MSLN expression did not significantly impact proliferation, TIL infiltration, or macrophage infiltration in omental tumor tissues. The accumulation of TILs, specifically B cells and CD8^+^ cells, in OvCa has been shown to increase survival. Furthermore, B-cell infiltration of omental metastases supports the development of an anti-tumor response [51,52]. Macrophages, specifically M-1-like macrophages, secrete chemokines and cytokines to recruit T cells to infiltrate the tumor leading to improved clinical response to therapeutics, an increase in the overall survival rate and a delay in recurrence [53].

In summary, our data support the conclusion that host MSLN expression plays a vital role in priming the tumor naïve peritoneal microenvironment consequently contributing to the progression of ovarian cancer metastasis, as deletion of host MSLN expression decreases the size of heterogeneous multicellular aggregates and ovarian cancer metastatic burden. Furthermore, ultrastructural changes to both the mesothelial cell surface and the sub-mesothelial matrix correlate with the reduction in tumor burden observed in MSLN^KO^ mice. These data support a role for host MSLN expression in the regulation of peritoneal tissue ultrastructure thereby impacting ovarian tumor metastatic success and provide support for further investigation of host MSLN as a target in ovarian cancer. The current study along with several preclinical and clinical studies demonstrate the significance of MSLN expression in both the host and tumor cells, making MSLN a promising target for OvCa treatment as current therapeutic effects seem moderate at best indicating further investigation is needed [12,54]. The discovery of novel MSLN dual-targeted drugs that could mimic the deletion of host MSLN expression, such as antibody-conjugates [55,56] and vaccines [57,58], and be cytotoxic to tumor cells would be ideal to improve survival in OvCa and other cancers with MSLN overexpression.

## 4. Materials and Methods

### 4.1. Reagents

Antibodies were purchased from several sources. Rabbit polyclonal proliferating cell nuclear antigen (PCNA; catalog # NBP1-40761) was purchased from Novus Biologicals. Rabbit C-ERC/Mesothelin (catalog # 28127) was purchased from Immuno-Biological Laboratories, Inc. Rat monoclonal F4/80 (catalog # ab6640) and CD45R (catalog # ab64100) were purchased from Abcam. All peroxidase-conjugated secondary antibodies (anti-rabbit IgG and anti-rat IgG) and peroxidase detection system reagents were from Vector Lab.

### 4.2. Mesothelin Mouse Model

MSLN wildtype (MSLN^WT^) and knockout (MSLN^KO^) mice were a gift from Dr. Ira Pastan (NCI/NIH, Bethesda, MD, USA) and were generated as previously described [13]. Briefly, embryonic stem cells from 129 SVJ mice were electroporated and transfected with the targeting plasmid pJMM10A linearized with *Not*I. Individual neomycin-resistant clones were picked, grown, and analyzed. DNA from two clones was injected into blastocysts from C57BL/6 mice to generate chimeric mice. Chimeric males were crossed with C57BL/6 females, and the offspring were analyzed for the mesothelin mutation. C57BL/6 heterozygous MSLN animals were intercrossed to generate homozygously mutated (knockout; MSLN^KO^) animals and wild-type littermate siblings (MSLN^WT^) to be used as control animals [13]. All animal procedures were carried out according to the regulations of the Institutional Animal Care and Use Committee (IACUC) at The University of Notre Dame. All murine studies were approved by the Institutional Animal Care and Use Committee, University of Notre Dame and were conducted in accordance with relevant guidelines and regulations of this committee.

### 4.3. Scanning Electron Microscopy (SEM) Tissue Processing and Analysis

Peritoneal tissue explants were put in primary fixative solution (2% Glutaraldehyde, 2% paraformaldehyde, 0.1 M Cacodylate buffer) for 8 h and washed 3 times in 0.1 M Cacodylate buffer (20 min. on a rocker). Secondary fixation was performed using 1% osmium tetroxide (OsO_4_) (diluted from 4% OsO_4_ stock solution with 0.1 M Cacodaylate buffer), followed by three 0.1 M Cacodylate buffer washes and three Milli-Q water rinses. Organs were then dehydrated in increasing ethanol concentrations (100 W for 40 s each) followed by critical point drying. Organs were mounted on carbon stubs (silver painted) and sputtered for SEM imaging using an FEI-Magellan 400 Field Emission scanning electron microscope (Notre Dame Integrated Imaging Facility, Notre Dame, IN, USA) [47,59]. SEM images were then analyzed by eye for notable ultrastructural features.

### 4.4. Second Harmonic Generation Microscopy

The omentum from MSLN^WT^ and MSLN^KO^ mice were dissected and placed between two microscope coverslips and placed on an Olympus FV1000 2-Photon Confocal Microscope. Using a 25× XLPlanN, 1.05 na water objective, omental collagen from the translucent opening was imaged using an excitation wavelength of 860 nm. Quantification of fibril orientation was performed using the ImageJ-based FibrilTool [60]. Statistical analysis was completed using Student’s *t*-test (GraphPad Prism).

### 4.5. Isolation and Propagation of Mesothelial Cells

Mesothelial cells from 3–6 month female MSLN^WT^ and MSLN^KO^ mice were isolated as described previously [47,61]. Briefly, MSLN^WT^ and MSLN^KO^ mice were injected with 0.125% trypsin/EDTA in the abdominal cavity and allowed to incubate on a warm surface for 20 min. To neutralize the trypsin, isolation medium composed of DMEM/F12 1:1, 10% FBS, 1% Pen/Strep was injected and collected. The abdominal cavity was washed once with isolation medium. The cells were centrifuged and red blood cells were lysed using ACK lysing buffer (Thermo Fisher Scientific, Waltham, MA, USA). Cells were centrifuged and resuspended in culture medium (DMEM/F12+GlutaMAX (Gibco), 15% FBS, 0.4 µg/mL hydrocortisone, 10 ng/mL EGF, 1% ITS (insulin, transferrin, selenium, 10 mM HEPES, and 1% penicillin/streptomycin. Cells were maintained at 37 °C, 5% CO_2_ in humid air.

### 4.6. Multicellular Aggregate Hanging Drop Culture

The mouse ovarian surface epithelial ID8 ovarian cancer cell line, syngeneic to immunocompetent C57BL/6 mice tagged with red fluorescent protein designated (ID8-RFP) were maintained as previously described [47,48,49,50,62,63]. Briefly, cells were maintained in Dulbecco’s Modified Eagle Medium (DMEM) supplemented with 4% fetal bovine serum (FBS), 1% penicillin/streptomycin, 5 µg/mL Insulin, 5 µg/mL transferrin and 5 ng/mL sodium selenite at 37 °C, 5% CO_2_ in humid air. Isolated MSLN^WT^ and MSLN^KO^ mesothelial cells were stained with CMFDA CellTracker dye and combined 1:1 (300 cells) with the mouse ovarian surface epithelial ID8-RFP tagged cells. As previously described, droplets (20 µL) were plated on the inner surface of a 150 × 25 mm tissue culture dish lid with phosphate-buffered saline in the lower dish [43,59]. Parallel monocultures of MSLN^WT^ and MSLN^KO^ mesothelial cells or mouse ovarian ID8-RFP cells were prepared as a control. MCA formation was confirmed and imaged using an Echo Revolve hybrid microscope. MCA size was measured using ImageJ and statistical analysis was completed using Student’s *t*-test (GraphPad Prism).

### 4.7. In Vivo Adhesion Assay

To evaluate early events of in vivo adhesion, MSLN^WT^ and MSLN^KO^ C57Bl/6 (*n* = 5) mice were injected i.p. with 10 × 10^6^ ID8-RFP cells and sacrificed the next day. Peritoneal adipose was excised, rinsed with PBS and imaged as described [48,63,64]. RFP signal was quantified using ImageJ. Statistical analysis was performed using Student’s *t*-test (GraphPad Prism). A *p*-value cutoff of 0.05 was counted as statistically significant.

### 4.8. Murine Allograft Model of Ovarian Cancer Metastasis

The mouse ovarian surface epithelial ID8 ovarian cancer cell line, syngeneic to immunocompetent C57BL/6 mice (MSLN^WT^ and MSLN^KO^), used in allograft tumor studies, were tagged with red fluorescent protein (RFP) and maintained as previously described [47,48,49,50,62,63]. To model the propagation and colonization events of ovarian cancer metastasis, ID8 RFP-tagged cells (7 × 10^6^ ) were i.p. injected into MSLN^WT^ and MSLN^KO^ mice. To monitor tumor progression, the mice were imaged once a week, beginning at 4 weeks post-injection, under isoflurane anesthesia, using the Bruker Xtreme In Vivo Imaging system. Additionally, mice were observed for signs of lethargy or ascites accumulation. The mice were euthanized for end-point dissection at 8 weeks post-injection. The ventral skin was pulled away and the peritoneal cavity was exposed with incisions down the midline and the sides of the ventral parietal peritoneum. The abdominal organs were scanned in situ as previously described using the Bruker Xtreme In Vivo Imaging system [48,63]. The organs were removed and imaged ex vivo. The abdominal and organ images underwent spectral unmixing using the Bruker Multispectral software as previously described [63]. Using ImageJ, tumor burden in the abdominal and organ images was analyzed by calculating the tumor area and the intensity of the RFP signal (Raw Integrated Density). To control for animal-to-animal differences in organ size the adjusted weight of the organs (weight^2/3^) was used [63]. Statistical analysis was performed using Student’s *t*-test (GraphPad Prism). A *p*-value cutoff of 0.05 was counted as statistically significant.

### 4.9. Histology

Abdominal organs were fixed in 10% formalin and processed for paraffin embedding for histological analysis. After deparaffinization, immunohistochemical analysis for MSLN (1:500), PCNA (1:200), F4/80 (1:50) and CD45R (1:800) was performed as previously described [48]. Slides were developed, counter-stained, dehydrated, and scanned into the eSlide Manager Database with the Aperio ScanScope CS (Leica Biosystems Inc., Buffalo Grove, IL, USA). Analysis was performed using the Aperio ePathology ImageScope pre-installed percent positive macro algorithm to quantitate the number of DAB chromogen positive (brown) cells. Statistical analysis was completed using Student’s *t*-test (GraphPad Prism).

## Data Availability

Not applicable.
